# Influence of polypharmacy on heart rate variability in older adults at the Hiroshima Atomic Bomb Survivors Recuperation Research Center, Japan

**DOI:** 10.1371/journal.pone.0209081

**Published:** 2018-12-12

**Authors:** Masahiro Okada, Kosuke Okada, Kohyu Fujii

**Affiliations:** 1 Department of Food and Dietetics, Hiroshima Bunka Gakuen Two-Year College, 3-5-1 Nagatsukanishi, Asaminami-ku, Hiroshima, Japan; 2 Department of Internal Medicine COOP Saeki Hospital, 3-11-29 Yahata-higashi, Saeki-ku, Hiroshima, Japan; Newcastle University, UNITED KINGDOM

## Abstract

**Background:**

Many studies have identified the risk of polypharmacy, but physiological evidence and methods of evaluation in these studies were poor. The relationship between polypharmacy and heart rate variability in older adults remains unclear. We investigated the relationship between polypharmacy in older adults, including atomic bomb survivors, and heart rate variability.

**Methods:**

We surveyed 56 older adults who did not need nursing care assistance in the Hiroshima Atomic Bomb Survivors Recuperation Center. Chronic diseases, types of medication, and lifestyle were assessed, and heart rate variability at rest was measured. We calculated heart rate variability indices including standard deviation of normal-to-normal RR intervals (SDNN), total power (TP), and very low frequency (VLF) and analyzed the relationship between the number of daily medication types and heart rate variability indices in older adults. The differences in heart rate variability indices were analyzed using six medications as a cut-off point.

**Results:**

Participants included 36 atomic bomb survivors and 20 non-atomic bomb survivors. The mean number of medication types was 3.6±3.4 (mean±standard deviation). SDNN, TP, and VLF decreased with an increased number of medications in all participants (*P*<0.01). When the standard of polypharmacy was set to more than six types of medications, SDNN, TP, and VLF were significantly lower in older adults who took six or more medications. Additionally, the mean number of medication types among atomic bomb survivors was higher than that of non-atomic bomb survivors (*P* = 0.008). The SDNN was significantly lower when atomic bomb survivors took six or more medications (*P*<0.001).

**Conclusions:**

We found that a lower heart rate variability in older adults, including atomic bomb survivors, is associated with polypharmacy. We showed physiological evidence of the influence of polypharmacy, which may be important for the healthy life expectancy and prognosis in older adults.

## Introduction

Medication is required to prevent and control chronic illness in older adults. Appropriate medication for older adults is desirable, but older adults are vulnerable to medication side effects [1, 2]. Although appropriate guidelines are shown for all medications, it is difficult to judge complex effects and side effects by prescription in older adults [3, 4]. Aging, an increase in chronic diseases, the number of hospitals involved in making a diagnosis, and an increase in the number of medications are associated with polypharmacy [5]. Polypharmacy increases the risk of adverse drug reactions and increases the risk of prescribing potentially inappropriate medications [6–9]. Polypharmacy may be associated with an increase in adverse events that are caused by drugs in older adults [10]. It has been reported that adverse events in older adults increase markedly when they are taking six or more medications [11]. It has also been reported that the fall risk for old adults increases with polypharmacy [12]. The risk of polypharmacy in older adults has been reported [13], but more detailed information is required.

Heart rate variability (defined as the beat-to-beat variation in heart rate intervals) reflects the balance of the autonomic nervous system [14]. Heart rate variability is influenced by various life stages and the person’s sex [15], and differences were recently shown even in short-term measurements, with a clear difference observed between young people and older adults. The influence of sex and age on heart rate variability disappears in older adults [16]. There are many reports that heart rate variability indices are effective for determining the prognosis of patients with myocardial infarction and heart failure or cancer [17–19]. The decrease in the standard deviation normal-to-normal RR intervals (SDNN) of the heart rate variability index is thought to be strongly related to the risk of mortality [20, 21]. It has also been suggested that heart rate variability is associated with a functional decline as frailty increases in older adults [22]. Ogliari et al. showed that a higher resting heart rate and lower heart rate variability are associated with a worse functional status and with a higher risk of future functional decline in older adults, independent of cardiovascular disease [23]. In particular, a decrease in SDNN for the heart rate variability index is thought to be important in the functional decline of older adults.

We hypothesize that polypharmacy may be related to the prognosis and future functional decline in older adults, and that heart rate variability indexes may be useful for predicting the influence of polypharmacy. However, there are few studies on the relationship between polypharmacy and heart rate variability in older adults. Therefore, we investigated the relationship between polypharmacy and heart rate variability in older adults at the Hiroshima Atomic Bomb Survivors Recuperation Research Center.

## Materials and methods

### Study participants

Fifty-six older adults who did not need nursing home assistance volunteered to participate in this study. The Human Studies Committees of Hiroshima Bunka Gakuen Two-Year College approved this study (Ethical Approval No: 29002). This study protocol was subsequently approved by the facility chief where the survey was conducted. The measurements were performed at the medical office at the Hiroshima Atomic Bomb Survivors Recuperation Research Center (Kanda Sansoh). Participants were daily users at the center who underwent rehabilitation and indoor moderate exercise, such as wading in a pool, pedaling on an ergometer, or using electrical massage. The measurement period was from December 2017 to March 2018. Verbal and written informed consent was obtained from all participants. All participants signed their own research consent form, and we strictly managed the data. All the participants were non-smokers, lived relatively independently, and were able to perform activities of daily living. All participants were not sick and were in good physical condition on the measurement day. Before performing all measurements, we confirmed via an interview, whether the participants were atomic bomb survivors (1945 atomic bombing); if they had a chronic disease; their movement frequency, sleep duration, and drinking frequency; and the number and type of medications they were taking. We verified the medications for all participants by looking at the medication notebook, and a doctor interviewed all participants and confirmed the details of the medications. We excluded participants with cardiac disorders and arrhythmias affecting heart rate variability. Older adults with dementia were also excluded from participating, as were those taking β-blockers [14].

### Measurement of heart rate variability

We measured heart rate variability between 13:00 and 15:00 in a quiet room. The room temperature was maintained at 26°C and participants wore light indoor clothes. Participants’ heart rate was measured in the resting state about 1 hour after lunch. Before measurement, we confirmed that the participants were not using a cardiac pacemaker. Heart rate and heart rate variability were measured and calculated using an SA-3000P device (Tokyo Iken Corporation, Tokyo, Japan) for short-term measurements (5 min). A power spectral analysis of the heart rate variability was used to determine the R-R interval, and the spectrum estimation was analyzed using fast Fourier transformation. Heart rate variability analysis was performed based on the guidelines of the Task Force of the European Society of Cardiology and the North American Society of Pacing and Electrophysiology [14]. Additionally, the heart rate variability analysis method was partially described in our previous publication [24]. SDNN is a time domain heart rate variability index; it is one of the most frequently used indexes and it is easily calculated using the normal-to-normal RR intervals [14, 25]. Spectral analysis is frequently the domain used for the heart rate variability index. Total power is a short-term estimate of the total power of the power spectral density in the range of frequencies between 0 and 0.40 Hz [14, 25]. This measure reflects overall autonomic activity, where sympathetic activity is a primary contributor. Spectral analysis is divided into very low frequency (VLF; 0.0033–0.04 Hz), low frequency (LF; 0.04–0.15 Hz), and high frequency (HF; 0.15–0.40 Hz) components. VLF is a prognostic marker for predicting cardiac events and it is a possible marker for adverse outcomes of any disease [14, 25]. Hormonal factors such as those in the renin–angiotensin system affect this spectral density. LF reflects the sympathetic activity that is modified by parasympathetic activity. HF reflects the parasympathetic activity and is influenced by respiration. The normalized low and high frequency (LF Norm and HF Norm) were the LF or HF power in normalized units: LF or HF / (Total power − VLF)×100. LF Norm and HF Norm were used to determine sympathetic and parasympathetic autonomic modulations, respectively [14]. We also calculated the LF-to-HF ratio, which reflects the balance between sympathetic and parasympathetic nervous system activities [14, 25].

### Definition of polypharmacy

In Japan, the adverse events risk for older adults taking six or more medications has been shown, and Japanese guidelines currently consider this to be the standard polypharmacy cut-off value [4, 11]. Based on the medication guidelines in Japan, we set six medications or more as cut-off for polypharmacy.

### Statistical analysis

All statistical analyses were performed using SPSS software (version 24.0, IBM SPSS Inc., Tokyo, Japan). Data for participant conditions and measurement are presented as the mean ± standard deviation (SD) or standard error (SE). Multiple linear regression analyses were undertaken to determine associations between the number of medications and heart rate variability in older adults. An unpaired *t*-test was used to compare the average number of medications for atomic bomb survivors and non-atomic bomb survivors. A one-way ANOVA was used to compare heart rate variability indices when medication use was classified into two groups (fewer than six, or six or more). *P* < 0.05 was considered significant.

## Results

Characteristics of the study population are shown in Table 1. The age (mean ± SD) of 56 older adults was 76.4 ± 5.7 years (range, 65–88 years). The participants comprised 41 females and 15 males, 36 atomic bomb survivors and 20 non-atomic bomb survivors, and 43 had chronic diseases and 13 had no chronic diseases. Mean height was 1.5 ± 0.1 m (range, 1.4–1.7 m), and weight was 57.3 ± 8.0 kg (range, 38.0–75.0 kg). The mean weekly movement frequency was 2.1 ± 1.9 days (range, 0–7 days), sleep duration was 6.7 ± 1.3 h (range, 4–10 h), and drinking frequency was 2.3 ± 2.8 days (range, 0–7 days). The mean number of medications taken that day was 3.6 ± 3.4 (range, 0–13). Participants had no life-threatening diseases and no participants were receiving anticancer drug treatment. Additionally, hypertension (28.6%), dyslipidemia (12.5%), and type II diabetes mellitus (10.7%) were frequent chronic diseases of the participants. For medications, drugs acting on the metabolic system (46.4%), drugs acting on the circulatory system (39.3%), drugs acting on the digestive system (37.5%), drugs acting on inflammation and immunity and allergy medications (32.1%), and drugs acting on the nervous system (26.8%) were frequently used. Herbal medicines and nutritional formulations were included in the number of medications.

**Table 1:** Characteristics of the study population (N = 56).

Characteristics	Number and values
Sex (Female:Male)	41:15
Atomic bomb survivor (Y:N)	36:20
Chronic disease (Y:N)	43:13
Age (years)	76.4 ± 5.7 (65–88)
Height (m)	1.5 ± 0.1 (1.4–1.7)
Weight (kg)	57.3 ± 8.0 (38.0–75.0)
Movement frequency (days/week)	2.1 ± 1.9 (0–7)
Sleeping duration (h)	6.7 ± 1.3 (4–10)
Drinking frequency (days/week)	2.3 ± 2.8 (0–7)
Medications (number/day)	3.6 ± 3.4 (0–13)

Values are presented as the mean ± standard deviation.

Range is shown in parentheses.

Relationships between heart rate variability and taking multiple medications were identified using multiple linear regression analysis (Table 2). A decreased SDNN interval was significantly associated with an increased number of medications in older adults (standard regression coefficient [β] = −0.478, *P* = 0.003). Decreased total power was significantly associated with an increased number of medications (β = −0.458, *P* = 0.004), and decreased VLF was significantly associated with an increased number of medications in older adults (β = −0.452, *P* = 0.003). Decreased low frequency was weakly associated with an increased number of medications (β = −0.346, *P* = 0.037). There was no relationship between other heart rate variability indices including heart rate and taking multiple medications in older adults.

**Table 2:** Relationship between heart rate variability and number of medications.

Variable	Mean ± SD	β (*P*)
Heart rate (beats/min)	72.3 ± 10.7	N
SDNN (ms)	25.8 ± 11.4	−0.478 (0.003)
Total power (ms^2^)	518.3 ± 508.8	−0.458 (0.004)
Very low frequency (ms^2^)	242.2 ± 269.9	−0.452 (0.003)
Low frequency (ms^2^)	154.2 ± 227.5	−0.346 (0.037)
High frequency (ms^2^)	121.9 ± 234.9	N
LF Norm	53.0 ± 22.3	N
HF Norm	47.0 ± 22.3	N
Low to high frequency ratio	2.0 ± 2.3	N

Values are given as mean ± standard deviation.

Analysis was performed after adjusting for sex, atomic bomb survivor, chronic disease, age, height, weight, movement frequency, sleep duration, and drinking frequency.

N, no significant influenced factors

β, standard regression coefficient

*P* values are shown in parentheses.

SDNN, standard deviation of normal-to-normal RR intervals

LF Norm, low frequency power in normalized units

HF Norm, high frequency power in normalized units

**Fig 1:**
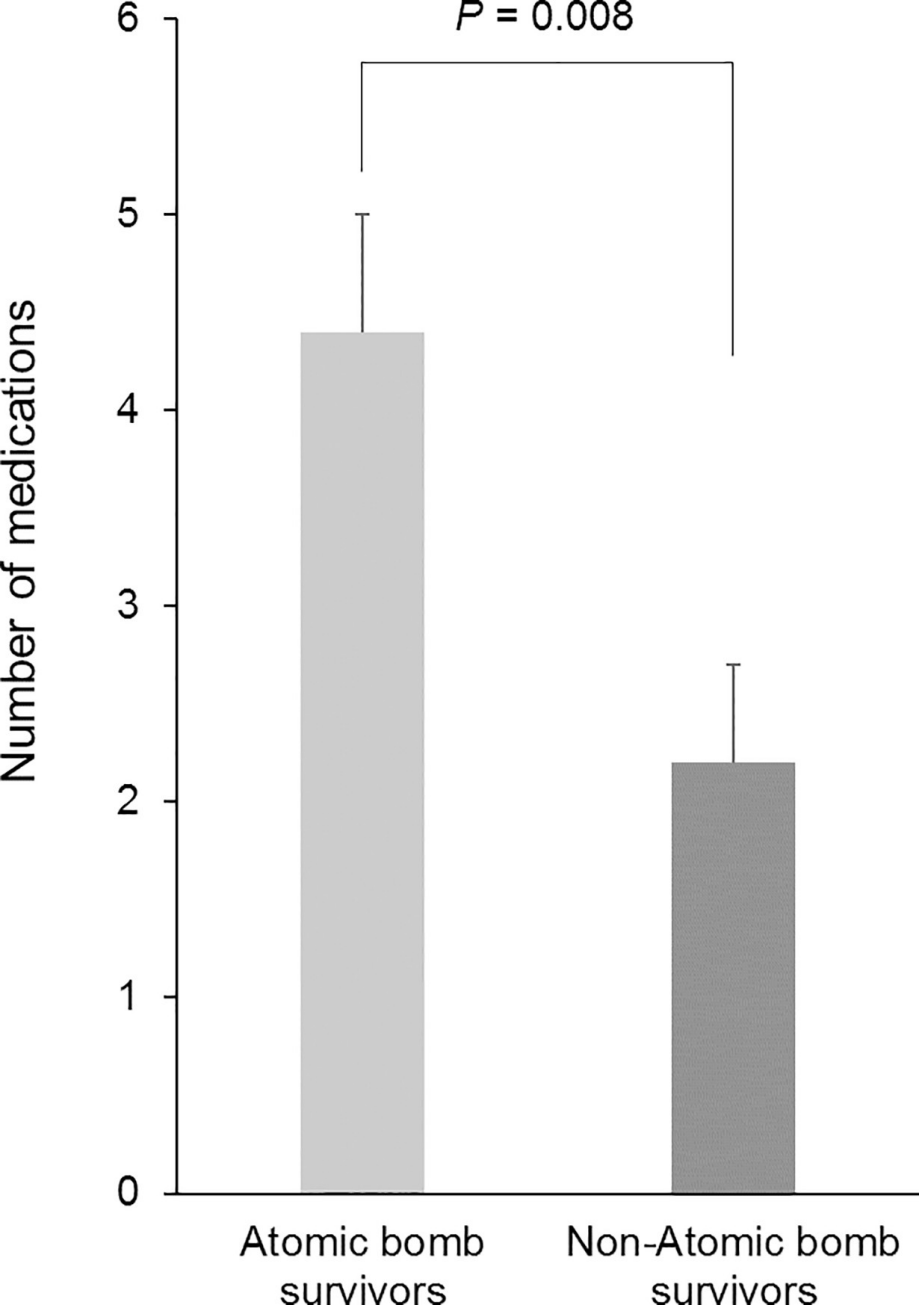
Difference in the number of medications between atomic bomb survivors and non-atomic bomb survivors. Values are presented as the mean ± standard error.

**Fig 2:**
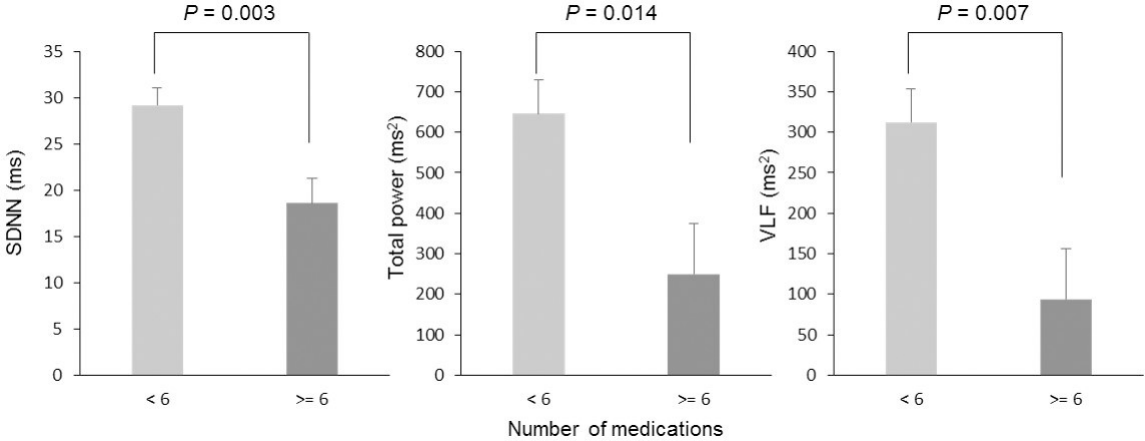
Difference in heart rate variability indexes when participants are classified as taking less than six (N = 38) or six or more (N = 18) medications. Heart rate variability indexes are the standard deviation of the normal-to-normal RR intervals (SDNN), total power, and very low frequency (VLF). Comparison analysis was adjusted by sex, atomic bomb survivor, chronic disease, age, height, weight, movement frequency, sleep duration, and drinking frequency. Values are presented as the mean ± standard error.

**Fig 3:**
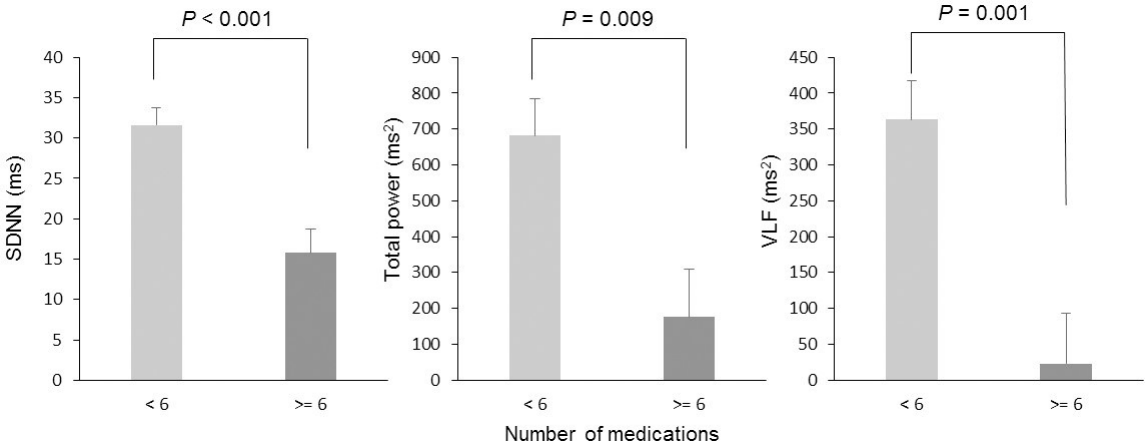
Difference in heart rate variability indexes when atomic bomb survivors are classified as taking less than six (N = 22) or six or more (N = 14) medications. Heart rate variability indexes are the standard deviation of normal-to-normal RR intervals (SDNN), total power, and very low frequency (VLF). Comparison analysis was adjusted by sex, chronic disease, age, height, weight, movement frequency, sleep duration, and drinking frequency. Values are presented as the mean ± standard error.

In Fig 1, we showed the average number of medications taken on a particular day compared between atomic bomb survivors and non-atomic bomb survivors. We found that the atomic bomb survivors were taking more medications compared with non-atomic bomb survivors (*P* = 0.008, 95% Cl, 0.616–3.818). In Fig 2, all participants were divided into groups of less than six medications and six or more medications, and the results of comparing heart rate variability indices are presented. There were 38 older adults taking less than six medications and 18 older adults who were taking six or more medications. There was a significant difference in SDNN (*P* = 0.003, 95% confidence interval [Cl], 3.690–17.372), total power (*P* = 0.014, 95% Cl, 85.469–707.952), and VLF (*P* = 0.007, 95% Cl, 63.148–374.577). Additionally, in Fig 3, the heart rate variability indices were compared by dividing atomic bomb survivors into less than six medications and six more medications. Among 36 atomic bomb survivors, 22 were taking less than six medications and 14 were taking six or more medications. The SDNN, total power, and VLF of participants taking six or more medications were significantly lower compared with participants taking fewer than six medications (SDNN: *P* < 0.001, 95% Cl, 7.810–23.742; total power: *P* = 0.009, 95% Cl, 140.620–872.159; VLF: *P* = 0.001, 95% Cl, 146.025–534.095).

## Discussion

Research on the influence of polypharmacy is important in older adults, but more physiological evidence regarding the risk of polypharmacy is required. To the best of our knowledge, the relationship between polypharmacy and heart rate variability in older adults has not been examined previously. The present study reports the results of our investigation of the relationship between polypharmacy and heart rate variability in older adults from December 2017 to March 2018 at the Hiroshima Atomic Bomb Survivors Recuperation Research Center.

The older adults who participated in our research at this facility were healthy or had a chronic disease, but did not need assistance. A study of nursing home residents suggested that polypharmacy was caused by the number of diseases and the use of multiple medical facilities [5]. The facility we examined was not a nursing home residence, but the same trend as that in the study involving the nursing home was seen in our study, and additionally, there was a correlation between atomic bomb survivors and polypharmacy [26]. We found that twice as many medications were taken by atomic bomb survivors compared with non-atomic bomb survivors. We suggest that the cause of increased polypharmacy among atomic bomb survivors may be the advancing age of the atomic bomb survivors, an increased number of concurrent diseases, and the tendency toward an increased number of prescription medications because people receive medical treatments at no cost. We investigated polypharmacy considering that Hiroshima may be a special environment that includes atomic bomb survivors.

We found that a higher number of medications per day decreases some heart rate variability indices in all participants. There are many previous studies showing that heart rate variability indices have been effective for determining life prognosis [17–19]. In particular, it is suggested that decreased SDNN is associated with a worse prognosis and an increased risk of mortality [20, 21]. Recent studies suggest that a decrease in the heart rate variability index, including SDNN, increases the risk of frailty and the risk of future functional decline in old age [23, 27]. Older adults with decreased TP and VLF may have a higher risk of frailty and reduced life expectancy associated with hip fracture [27, 28]. We found that an increased number of medications is strongly associated with a decreased SDNN, TP, and VLF. Reduced SDNN in older adults with chronic diseases is likely to be a major risk, such as for a worse prognosis. Additionally, the risk of mortality in older adults may be increased by frailty and functional decline [29, 30]. In older adults, higher levels of inflammation factors such as C-reactive protein (CRP) and insulin resistance were associated with lower SDNN and VLF [31, 32]. In older adults, polypharmacy may continue to influence daily heart rate variability, and increase the risk of pathogenesis in the future. Additionally, this result of heart rate variability was considered to be influenced by the type of chronic disease such as type II diabetes mellitus and multiple chronic diseases in participants [33]. Although we did not show detailed results, we found a relationship between reduced heart rate variability and an increased number of medications even after adjusting for the participant’s specific chronic disease or multiple diseases. We suggest that it is important to control the onset of chronic diseases with medication, but considering the number of medications and the effects of the medications is also important in older adults. Adverse drug reactions were shown to correlate with the use of more medications with different schedules in older adults [34, 35]. We also found a strong association between the number of medications and heart rate variability indexes. Although this change in heart rate variability with polypharmacy may be related to the influence of adverse drug reactions, chronic diseases seemed to be well controlled in the participants in this research. Heart rate variability may be a marker for the influence of invisible polypharmacy, and it may contribute to the prognosis and healthy life expectancy of older adults.

We also divided the participants into categories of six or more medications and less than six medications and compared heart rate variability in these groups. In studying the polypharmacy cut-off point, some studies reported that the risk of falls and frailty increases when older adults take five or more drugs [12, 36]. In a recent study in Japan, it was reported that adverse events related to medications increased when older adult patients were taking six or more medications [11]. We found that there was a large difference in heart rate variability in older adults taking six or more medications, and our results may be consistent with the polypharmacy situation in Japan. The heart rate variability indexes SDNN, TP, and VLF in older adults taking six or more medications were significantly lower compared with older adults taking less than six medications. Additionally, to investigate the influence of polypharmacy in atomic bomb survivors, a comparative analysis was also conducted within the atomic bomb survivor group. There was a large difference within the group of atomic bomb survivors who were taking six or more medications compared with those taking less than six medications. In particular, SDNN was lower in participants taking six or more medications compared with those taking less than six medications in the atomic bomb survivor group. Thus, the heart rate variability index SDNN may be the best method to examine the influence of polypharmacy in older adults. For atomic bomb survivors who experience polypharmacy, these results are concerning. Okada reported a reduced number of drugs and a reduction in the number of falls in older adults including atomic bomb survivors, but research of this nature is rare [26, 37]. The older adults in Hiroshima include many atomic bomb survivors. We suggest that reducing multiple medications may be necessary for a longer healthy life expectancy for atomic bomb survivors.

The results of this study suggest that the influence of polypharmacy with a cut-off point of six medications can be reflected by heart rate variability indexes in older adults. However, further research, such as investigating the effect of reversing the lowered heart rate variability by reducing the number of medications, is necessary. Additionally, to accurately discuss prognosis and mortality, a participant follow-up survey is required.

There are some limitations to our research. First, this study is an observational study and the sample size is small. Therefore, we were unable to analyze detailed adverse drug reactions and discuss mechanisms of lower heart rate variability. More information is required to reduce the adverse drug reactions in older adults and the risk of iatrogenic illness, including polypharmacy [2]. We suggest that research using a larger sample size and research including clinical studies are required to increase the credibility of the heart rate variability index. Second, the older adults who participated in this study were relatively healthy, and they had controlled the onset of chronic disease, with no need for nursing care. Additionally, the participants were not taking anticancer or similar types of drugs. In hospitals and nursing homes, there are more confounding factors that affect the heart rate variability in older adults, and research in older adults needs to consider various stages of care that is required. We also believe that it is necessary to consider polypharmacy for each chronic disease such as diabetes [33]. Third, the heart rate variability measurement used in our research was obtained using one device and analyzed for specific indices in the short term. Although the burden on the older adults should be taken into consideration, it is necessary to further verify heart rate variability indices using various devices. Fourth, although the current medication situation of the participants was known, we did not consider medication history in our data. It may also be important to investigate the period of polypharmacy to determine the detailed influence on heart rate variability. Fifth, there were no restrictions on food quantity and composition at the meal (lunch) that was eaten before measurements were taken. Given the physical burden of older adults, strong restrictions on food intake would be difficult for the participants. Additionally, our research was conducted in Hiroshima, and it may be necessary to think about differences in this area. Thus, more studies are required to standardize and validate our results. Regardless of the limitations, our results represent a new finding and it is important information when considering polypharmacy in older adults.

## Conclusions

Polypharmacy may cause serious problems for the health and prognosis of older adults. Polypharmacy increases the inappropriate use of medications and increases the risk of adverse drug reactions. Studies on polypharmacy are also important for the healthy life expectancy of older adults including atomic bomb survivors. Many studies have investigated the risk of polypharmacy, but the physiological evidence and methods of evaluation were poor in those studies. In this study, we examined the influence of polypharmacy using the heart rate variability index in older adults. In the future, we may be able to evaluate the influence of polypharmacy using the heart rate variability index, which would be particularly important for older adults. Although our results examined one physiological index and one evaluation method, we believe that this result is important for advancing research on polypharmacy.
